# Juvenile pemphigus vulgaris: A narrative review

**DOI:** 10.1097/MD.0000000000042611

**Published:** 2025-05-23

**Authors:** Shyamkumar Sriram, Mambakkam Jayakanth, Tanveer Alam, Shazina Saeed, Shamimul Hasan

**Affiliations:** a Department of Rehabilitation and Health Services, College of Health and Public Service, University of North Texas, Denton, TX; b Department of Internal Medicine, Patiala Heart Institute, Patiala, India; c Department of Biomedical Dental Sciences, College of Dentistry, Al Baha University, Alaqiq, Al Baha, Kingdom of Saudi Arabia; d Amity Institute of Public Health & Hospital Administration, Amity University, Noida, Uttar Pradesh, India; e Department of Oral Medicine and Radiology, Faculty of Dentistry, Jamia Millia Islamia, New Delhi, India.

**Keywords:** autoimmune, histopathology, juvenile pemphigus vulgaris, pemphigus, vesiculobullous disease

## Abstract

Pemphigus vulgaris is a chronic autoimmune dermatosis that primarily affects the skin and mucous membranes. Juvenile pemphigus vulgaris (JPV), a pediatric variant of pemphigus vulgaris, displays unusual features that may mimic other mucosal lesions. This similarity leads to diagnostic and therapeutic difficulties, often causing delays in diagnosis. Primary treatment consists of systemic corticosteroids and immunosuppressive agents, with biological therapies, such as rituximab, emerging as potential alternatives. Timely diagnosis and treatment are essential to improve prognosis and reduce complications related to long-term immunosuppressive therapy. A comprehensive electronic and manual literature search was conducted using various databases to consolidate the existing literature on JPV, focusing on its epidemiology, pathogenesis, clinical presentation, diagnosis, treatment options, and long-term outcomes in pediatric patients. A detailed review focusing on JPV has been conducted over the last 10 years (2014–2024). A literature review identified 37 cases of JPV in 16 studies. Owing to the rarity of JPV, much of the available evidence relies on case reports and case series, resulting in poorly defined response rates to different treatment options. Additionally, long-term monitoring of patients with disease recurrence is lacking. By identifying gaps in the current knowledge and offering a comprehensive overview, this review seeks to enhance awareness and guide future research and management strategies for JPV.

## 1. Introduction

Autoimmune bullous diseases are classified into 2 groups based on the depth of blistering: pemphigus disease, characterized by intraepithelial blistering (blisters forming within the epithelium), and pemphigoid disease, marked by subepithelial blistering (blisters forming beneath the epithelium). Both groups included multiple subtypes with distinct clinical and diagnostic characteristics.^[1]^

Pemphigus is an autoimmune, intraepithelial blistering disorder that affects mucocutaneous membranes. It occurs due to immunoglobulin G autoantibodies targeting desmoglein 1 and 3, which activate a protease that disrupts cell adhesion among epidermal keratinocytes at the stratum spinosum level. This leads to acantholysis, resulting in the formation of potentially life-threatening bullae and erosions.^[2–4]^ In contrast, pemphigoid results from autoantibodies attacking key proteins in the basement membrane zone, such as BP180 (type XVII collagen) and BP230, which play a crucial role in anchoring basal keratinocytes to the basement membrane. This autoantibody interaction activates the complement system and recruits inflammatory cells, ultimately causing subepidermal blister formation.^[5]^

Several clinical subtypes of pemphigus have been recognized, each exhibiting varying degrees of cutaneous and mucous membrane involvement. These subtypes include Pemphigus vulgaris (PV), pemphigus vegetans, pemphigus erythematosus, pemphigus foliaceus, pemphigus herpetiformis, IgA pemphigus, and paraneoplastic pemphigus. PV, the most common variant, accounts for 70% to 80% of cases. It is characterized by flaccid blisters that can form on any part of the skin along with painful erosions of the mucous membranes, which may serve as the initial sign of the disease.^[2,6,7]^

Although PV is classified as an autoimmune dermatosis, the exact mechanism underlying the loss of desmosomal integrity after autoantibody binding remains poorly defined. Several theories have been proposed to explain the underlying mechanisms, including desmoglein compensation theory, steric hindrance theory, multiple hits hypothesis, antibody-induced apoptosis, and signaling theory. However, none of these theories have led to definitive conclusions.^[6,7]^

Environmental factors that may trigger immune dysfunction and lead to exacerbation of PV include dietary components (such as garlic, red wine, and peppers), medications (including penicillamine, captopril, nonsteroidal anti-inflammatory drugs, and antibiotics), viral infections, ultraviolet radiation, stress, ionizing radiation therapy, allergens, and pesticides.^[8,9]^ Brenner et al revealed a higher risk of PV associated with pesticide exposure and occupational exposure to metal vapor. Smoking appeared to have a protective effect against PV, possibly due to its impact on the immune system and hormonal factors.^[10]^

With an incidence rate of 0.1 to 0.5 per 1,00,000 individuals, PV mainly affects adults, particularly those in their 4th to 6th decades, while children and the elderly are rarely affected.^[11]^

Owing to the rare occurrence of autoimmune bullous diseases in the pediatric population, there is a lack of literature on their prevalence, clinical characteristics, and treatment response.^[12]^ Around 1.4% to 3.7% of all PV cases are found in individuals aged 18 years or younger. Pediatric PV is categorized as pediatric/childhood PV, affecting those under 12, and juvenile/adolescent PV, affecting individuals between 12 and 18. Most pediatric pemphigus cases are of the vulgaris type, often manifesting around the age of 12 years.^[6,7,13]^

Pediatric pemphigus presents significant diagnostic and therapeutic challenges, often displaying atypical clinical and histological characteristics that frequently result in delayed diagnosis.^[14]^

There are no Food and Drug Administration sanctioned treatment guidelines for pediatric PV, and the current treatment strategies lack robust evidence owing to the lack of controlled trials conducted in this population. Currently, there are no established treatment guidelines for pediatric PV.^[7,15]^ Usually, the primary treatment for pemphigus consists of high-dose systemic corticosteroids (sCS),^[14]^ while adjuvant therapies, such as azathioprine (Aza), cyclophosphamide (Cyp), dapsone (Dap), and mycophenolate mofetil (MMF), are employed to enhance treatment response and reduce corticosteroid (CS) dependency.^[7]^

Recent progress in understanding disease pathogenesis has paved the way for the development of innovative targeted therapies such as rituximab (RTX). RTX is a monoclonal antibody that specifically targets CD20-positive B lymphocytes, and has demonstrated remission in refractory pediatric and adult PV cases.^[16]^ JPV has a highly variable prognosis, although it is generally regarded as more favorable than that in adults.^[15]^

This narrative review aimed to synthesize the existing literature on JPV, focusing on its prevalence, clinical presentation, diagnosis, and management in the pediatric population.

## 2. Materials and methods

This review followed the Preferred Reporting Items for Systematic Literature Reviews and Meta-Analyses 2020 guidelines for comprehensive search strategy and methodology.

A detailed electronic and manual literature search was conducted using PubMed and Scopus databases. The Google Scholar search engine was also used to ensure the comprehensiveness of the search and to identify any gray literature. The search used a combination of the following Medical Subject Headings terms: “Pemphigus Vulgaris”[Mesh] AND “Pediatrics”[Mesh] OR (“Child”[Mesh] OR “Juvenile”[Mesh] OR “Adolescent”[Mesh]). The research question was defined by PICO as follows:

Participants (P): Patients between 12 and 18 years of age with a confirmatory diagnosis of PV through histopathological or immunofluorescence (IF) studies.

Interventions (I): CS, RTX, and other immunosuppressive therapies.

Comparator (C): There was no control taken due to the scarcity of clinical trials.

Outcomes (O): Long-term adverse effects, remissions, and relapse.

The following inclusion and exclusion criteria were considered.

Inclusion criteria

1. Patients between 12 and 18 years of age with a confirmatory diagnosis of PV through histopathological or IF studies.
2. Full-text articles published in English language within the last 10 years (2014–2024).
3. Case reports, case series, prospective or retrospective cohort studies, case-control studies, and randomized controlled trials.

Exclusion criteria

1. Patients below 12 years and above 18 years of age, or other subtypes of pemphigus.
2. Articles published before 2014 and in languages other than English.
3. Reviews, personal opinions, conference proceedings, and letters to the editor.

The titles and abstracts of the retrieved articles were systematically evaluated by 2 independent authors. This dual review process aimed to minimize bias and ensure the relevance of the selected studies. In cases where discrepancies arose between the 2 reviewers, a 3rd author resolved these differences through discussion and consensus. To further enhance the comprehensiveness of the review, a manual evaluation of the references cited in all the retrieved articles was conducted. This step was crucial for identifying any additional pertinent studies that may have been overlooked during the initial electronic search.

Relevant data, including demographic information, clinical features, diagnostic criteria, treatment modalities, and outcomes, were extracted from the selected articles. The extracted data were then qualitatively analyzed to identify common themes, gaps in the literature, and areas for future research.

## 3. Results

**Figure 1.**
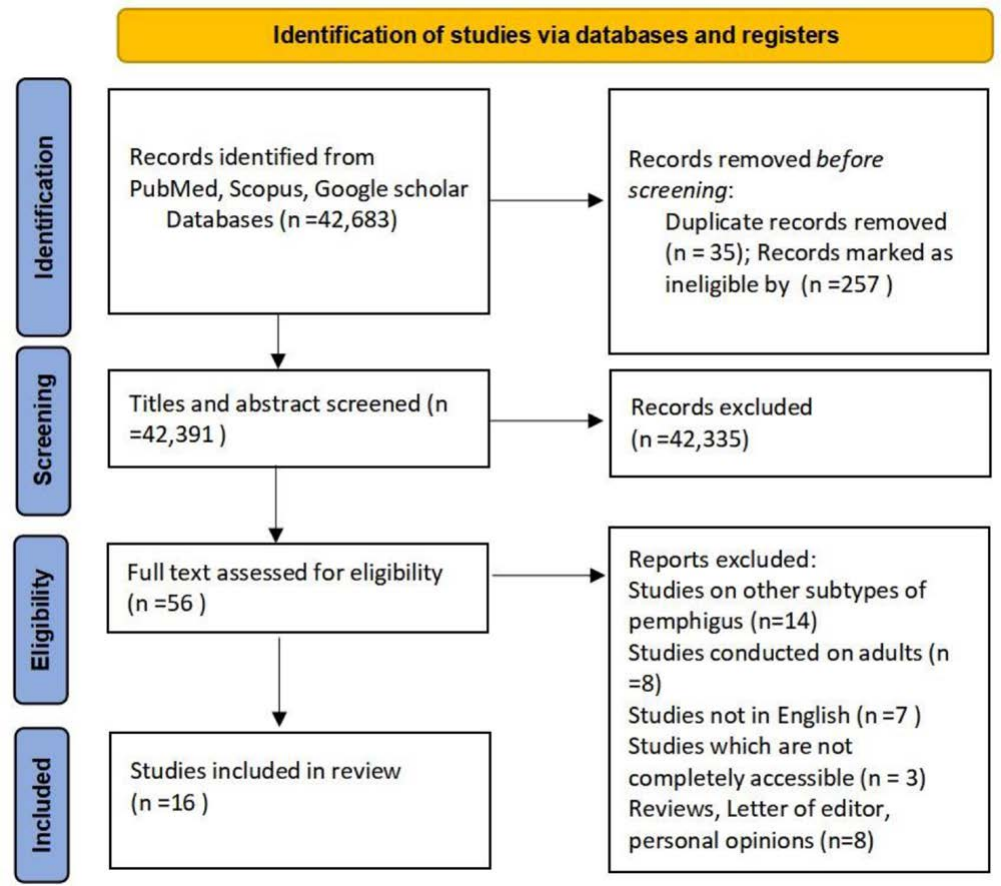
PRISMA flowchart. PRISMA = Preferred Reporting Items for Systematic Literature Reviews and Meta-Analyses.

A comprehensive literature review identified 37 cases of JPV across 16 studies, as depicted in the PRISMA flowchart (Fig. 1). The reviewed literature, summarized in Table 1, provided insights into the study design, country of origin, age, sex, affected sites, clinical presentation, diagnostic and therapeutic approaches, and patient outcomes.^[6,12,14,16–28]^

**Table 1:** Summary of the reported juvenile pemphigus vulgaris cases.

S. No.	Author & year	Study design	Country	No. of patients	Age/sex	Site	Clinical features	Diagnostic Aid	Treatment	Follow-up/relapse
1	Haberland C et al, 2014^[17]^	Case report	Latin America	1	17/F	Oral cavity	Hyperplastic gingiva	HP, DIF and ELISA -ve anti‐dsg 1 and anti‐dsg 3 Abs	Surgical excision	NA
2	Vinay K et al, 2014^[18]^	Retrospective analysis	India	5	17/M	Mucocutaneous	NA	HP, DIF and ELISA + for anti‐dsg 1 and anti‐dsg 3 Abs	CS, Aza, Dap & MMF refractory; followed by RTX therapy	Complete remission off therapy
					17/M	Mucocutaneous	NA	HP, DIF and ELISA + for anti‐dsg 1 and anti‐dsg 3 Abs	CS refractory. CSs adverse effect-iatrogenic cushing. Later, treated with RTX	Complete remission on therapy
					17/F	Mucocutaneous	NA	HP, DIF and ELISA + for anti‐dsg 1 and anti‐dsg 3 Abs	Refractory to CS; treated with RTX	Complete remission off therapy
					13/F	Mucocutaneous	NA	HP, DIF and ELISA + for anti‐dsg 1 and anti‐dsg 3 Abs	CS & Aza refractory. Later, treated with RTX	Complete remission off treatment
					12/M	Mucocutaneous	NA	HP, DIF and ELISA + for anti‐dsg 1 and anti‐dsg 3 Abs	CS & Aza refractory. Later, treated with RTX	Control of disease activity
3	Buch JY et al, 2016^[19]^	Case series	India	2	12/M	Mucocutaneous	Fluid filled scalp, skin and oral lesions	TS and HP	1 cc IV DM BD & Aza OD × 20 d. Later, DAP therapy was given.	Complete remission on treatment
									RTX was given due to adverse effects of DAP. 300mg RTX infusion for 5–6 h, then 2nd dose after 15 d.	
					12/M	Mucocutaneous	Fluid filled scalp, skin and oral lesions	TS and HP	I.V DM (1 cc) once a day, gradually tapered over 30 d. Later, RTX was given.	Complete remission on treatment
4	Srivastava A et al, 2017^[20]^	Case report	India	1	14/M	Mucocutaneous	Diffuse erosions on the buccal and labial mucosa, lateral and ventral tongue	IIF	Topical use of 0.1% TCA with analgesic and anesthetic. Later, RTX with aloe vera gel was given.	Ongoing follow-up.
5	Salman A et al, 2017^[12]^	Retrospective analysis	Turkey	2	14/M	Mucocutaneous	Diffuse erosions on the buccal mucosa	HP and IF	Refractory to MP, Aza, Dap & IV IG. Later, RTX was given.	Complete remission on treatment
					16/M	Oral cavity	Diffuse erosions on the buccal mucosa	HPand IF	Refractory to MP, Aza, MMF & IV IG. Later, RTX was given.	Complete remission off treatment
6	Gupta J et al, 2017^[21]^	Prospective study	India	2	12/M	NA	NA	HP, DIF and ELISA + for anti‐dsg 1 and anti‐dsg 3 Abs	Refractory to CS & Aza; followed by RTX therapy.	Complete remission off treatment
					12/M	NA	NA	HP, DIF and ELISA + for anti‐dsg 1 and anti‐dsg 3 Abs	Refractory to CS & Cyp; followed by RTX therapy.	Complete remission off treatment
7	Surya V. et al, 2018^[22]^	Case report	India	1	12/M	Oral cavity	Diffuse erosive lesions on the buccal & labial mucosa, tongue ventral, and floor of mouth	TS showed acantholytic cells	Short course of topical CSs	Complete remission on treatment
8	Hettiarachchi KS et al, 2018^[23]^	Case report	Sri Lanka	1	15/M	Mucocutaneous	Scar lesions on the face, scalp, and back; erosive lesions on buccal mucosa & tongue	HP and IF	A week therapy of 10 mg Pred (in tapering dose) along with a mouthwash	Responsive to CSs. Referred to dermatologist for skin lesions.
9	Bilgic-Temel A et al, 2019^[16]^	Retrospective analysis	Turkey	3	13/M	Mucocutaneous	Multiple flaccid vesicles and erosions on the trunk, extremities, and oral mucosa	HP, IF and ELISA	Refractory to 3 months MMF, 8 months Aza, 2 months Dap, & 9 months MP therapy. Later, treated with RTX for 42 months.	Partial response off therapy
					14/M	Mucocutaneous	Multiple flaccid vesicles and erosions on the trunk, extremities, and oral mucosa	HP, IF and ELISA	Refractory to 7 months MP, 2 months Aza, 7 months MMF, & 6 months IV IG therapy. Later, treated with RTX for 34 months.	Partial response off therapy
					16/M	Mucocutaneous	Multiple flaccid vesicles and erosions on the trunk, extremities, and oral mucosa	HP, IF and ELISA	Refractory to 24 months MP, & 12 months Aza therapy. Later, treated with RTX for 9 months	Complete remission off therapy
10	Broshtilova V et al, 2019^[24]^	Case report	Romania	1	14/F	Mucocutaneous	Multiple erosions, oval blisters covered with hemorrhagic crusts and flaccid blisters and erosions on the trunk, extremities; erosions on the hard palate	HP, DIF and IIF	MP refractory; CSs therapy exhibited adverse effects; Dap 25 mg/d was tried without any improvement. Later RTX was given.	Complete remission
11	Sakhiya J et al, 2020^[25]^	Retrospective, non-randomized, single-center open case series	India	1	17/M	Mucocutaneous	NA	HP, IF and ELISA	Pred & Aza refractory; later RTX was given	Complete remission off therapy
12	Kianfar N et al, 2022^[26]^	Retrospective, single-center study	Iran	9	14/F	Mucocutaneous	NA	HP, DIF, ELISA	Pred & Aza refractory; CSs therapy exhibited adverse effects; later RTX was given	NA
					14/M	Mucocutaneous	NA	HP, DIF, ELISA	refractory to Cs, MMF, Aza, therapy, later RTX was given	NA
					16/F	Mucocutaneous	NA	HP, DIF, ELISA	Pred refractory; later RTX was given	NA
					14/M	Mucocutaneous	NA	HP, DIF, ELISA	Pred refractory; later RTX was given	NA
					16/F	Mucocutaneous	NA	HP, DIF, ELISA	Pred refractory; later RTX was given	NA
					17/M	Mucocutaneous	NA	HP, DIF, ELISA	Pred & Aza refractory; later RTX was given	NA
					17/F	Mucocutaneous	NA	HP, DIF, ELISA	Pred refractory; later RTX was given	NA
					16/F	Mucocutaneous	NA	HP, DIF, ELISA	Pred, Aza & MMF refractory; later RTX was given	NA
					16/F	Mucocutaneous	NA	HP, DIF, ELISA	Pred refractory; later RTX was given	NA
13	Mistry BD et al, 2022^[27]^	Case report	Canada	1	13/M	Mucocutaneous	Multiple erosions on the buccal mucosa, tongue and palate	HP	Systemic CSs and RTX	No recurrence within a 2-year follow-up
14	Santiago-Vázquez M et al, 2022^[28]^	Case report	Latin America	1	14/F	mucocutaneous	Multiple flaccid vesicles and erosions on the trunk, extremities, and oral mucosa	HP	Oral CSs (0.5 mg/kg/d), IV CSs (MP 40mg), IV IG treatment. Later, RTX therapy was given.	Recovered after 18-month therapy; No relapse.
15	Hasan S et al, 2024^[6]^	Case report	India	1	16/F	Oral mucosa	Multiple erosions on the buccal mucosa, and tongue	HP, IF, and ELISA	Topical steroids-Tab. Betnesol 1 mg, turbocort paste, vitamin C tab. & vitamin D oral solution.	Complete resolution after 3 weeks of topical steroid therapy. No recurrences in 1 year follow-up.
16	Renert-Yuval Y et al, 2024^[14]^	Retrospective study	Israel	5	13.5/M	Oral mucosa	Multiple erosive lesions on buccal mucosa & tongue	HP and IF	Ongoing systemic CSs with RTX as a 2nd-line therapy.	Recurrence after a follow-up of 97.6 months.
					12/M	Oral mucosa	Multiple erosive lesions on buccal mucosa & tongue	HP and IF	20 months of systemic CRs with Dap.	Complete remission after 19.3months follow-up
					12/F	Mucocutaneous	Multiple erosive lesions on skin, buccal mucosa & tongue	HP and IF	42 months of systemic CSs, RTX was later used as a 2nd-line therapy	Complete remission after 191.7months follow-up
					15/F	Mucocutaneous	Multiple erosions on skin, buccal mucosa, tongue & palate	Histopathology and IF	60 months of systemic CSs, RTX was later used as a 2nd-line therapy	Complete remission after 68.6 months follow-up.
					16.6/M	Mucocutaneous	Multiple erosions on skin, buccal mucosa, tongue & palate	HP and IF.	Treated with Dap. & MMF	Complete remission after 4 months follow-up

Aza = azathioprine, CS = corticosteroids, Cyp = cyclophosphamide, Dap = dapsone, DIF = direct immunofluorescence, DM = dexamethasone, ELISA = enzyme-linked immunosorbent assay, HP = histopathology, IF = immunofluorescence, IIF = indirect immunofluorescence, IV IG = intravenous immunoglubulin G, MMF = mycophenolate mofetil, MP = methylprednisolone, Pred = prednisolone, RTX = rituximab, TCA = triamcinolone acetonide, TS = Tzanck smear.

This review included 8 case reports,^[6,17,20,22–24,27,28]^ 6 retrospective studies,^[12,14,16,18,25,26]^ 1 prospective study,^[21]^ and 1 case series.^[19]^ A total of 37 patients were affected, consisting of 23 males (62.2%) and 14 females (37.8%), with ages ranging from 12 to 17 years. The cases included 13 patients from India,^[6,18–22,25]^ 9 from Iran,^[26]^ 5 each from Israel^[14]^ and Turkey,^[12,16]^ 2 from Latin America,^[17,28]^ and 1 each from Sri Lanka, Romania, and Canada.^[23,24,27]^

Most patients presented with mucocutaneous involvement (n = 29; 78.4%),^[12,14,16,18–20,23–28]^ while exclusive oral involvement was noted in 6 patients (16.2%).^[6,12,14,17,22]^ The site of involvement was not reported in 2 cases (5.4%).^[21]^ Multiple, thin-walled vesicles, blisters, and erosions affecting the scalp, skin, and extremities were the most common findings. Clinical features were not documented in seventeen patients.^[18,21,25,26]^ Oral lesions appeared as flaccid vesicles and erosions, primarily affecting the buccal mucosa, labial mucosa, tongue, floor of the mouth, and palate. Hyperplastic gingiva as the sole manifestation of JPV was reported in 1 patient.^[17]^

The diagnosis was primarily based on Tzanck smear (TS), histopathology (HP), IF, and serological tests (enzyme-linked immunosorbent assay [ELISA]). Histopathological examination frequently confirmed acantholysis and suprabasilar splits within the epithelium. Direct immunofluorescence often revealed intercellular immunoglobulin G and C3 deposits, while ELISA tests occasionally detected PV-specific antibodies.

In this review, diagnosis was established using HP, IF, and ELISA in 23 patients,^[6,16–18,21,24–26]^ HP and IF in 8 patients,^[12,14,23]^ and TS combined with HP in 2 patients.^[19]^ Additionally, the diagnosis was based solely on HP in 2 patients,^[27,28]^ IF in 1 patient,^[20]^ and TS in 1 patient.^[22]^

Initial treatment included sCS,^[12,14,16,18,19,21,23–28]^ Aza,^[12,16,18,19,21,24–26]^ MMF,^[12,14,16,18,26]^ Dap,^[12,14,16,18]^ and intravenous immunoglobulin (IV IG).^[12,16,28]^ In some cases, topical steroids,^[6,20,22]^ and adjunctive therapies such as aloe vera gel^[20]^ or vitamin supplements^[6]^ were used. Surgical excision of the hypertrophic gingiva was performed in 1 case report.^[17]^

However, many patients were refractory to these treatments. RTX emerged as the most effective therapy for refractory cases, often leading to complete or partial remission by the final follow-up visit.^[12,14,16,18–21,24–27]^ Control of disease activity was reported in 1 case.^[28]^ A few studies documented adverse effects, including infusion reactions,^[18,21]^ angioedema,^[18]^ herpes zoster infection,^[21]^ chills, fever, dyspnea, rigor, tachycardia,^[26]^ and dental abscess.^[12]^

## 4. Discussion

PV often presents diagnostic and therapeutic challenges, with therapeutic difficulties being even more pronounced in pediatric patients. Furthermore, there is a dearth of studies that provide long-term evidence of the progression of pediatric PV.^[14]^

Adolescent/juvenile PV (JPV) affects individuals aged 12–17 years old.^[13,18,24,29]^ It equally affects males and females in contrast to slight female predominance in adult PV.^[16,22]^ Our review findings are consistent with those in the existing literature. In our review, 23 males and 14 females were affected, with the youngest patient being 12 years old and the oldest 17 years old.

PV demonstrates a strong genetic and environmental predisposition and is more frequently observed in Ashkenazi Jews, Japanese individuals, and individuals of Mediterranean descent.^[4]^ Our review findings aligned with the published literature, with patient distribution as follows: 13 patients from India,^[6,18–22,25]^ 9 from Iran,^[26]^ 5 each from Israel^[14]^ and Turkey,^[12,16]^ and 2 from Latin America.^[17,28]^ Additionally, 1 patient each was reported from Sri Lanka,^[23]^ Romania,^[24]^ and Canada.^[27]^

The clinical presentation of JPV is similar to adult PV presenting with flaccid vesicles and blisters of both skin and mucosal surfaces, which on rupturing produces ulcers and erosions and subsequently crusted lesions. These lesions can further show the cutaneous involvement of the face, trunk, breast, back, and groin areas.^[22]^ The reviewed patients predominantly exhibited mucocutaneous involvement and presented as multiple, flaccid vesicles and erosions affecting the scalp, back, skin, and extremities.^[6,14,20,23,27]^

About 80% to 90% of PV patients exhibit oral manifestations at some stage of the disease. However, in ≥60% of patients, these lesions may be the sole presenting symptom. Oral lesions initially present as vesiculobullous and are prone to rupture, resulting in the formation of new flaccid bullous lesions as the older ones ulcerate. Erosive and ulcerative lesions are the hallmark features of PV.^[30]^ Oral PV typically affects areas subjected to mechanical trauma, making the buccal mucosa the most common site, followed by the palatal and lingual mucosa.^[31]^ Oral lesions exhibit a positive Nikolsky sign, where a lack of epidermal cohesion at the dermal-epidermal junction causes lateral blister expansion with gentle pressure or rubbing. Oral lesions may also show a positive Asboe-Hansen sign (or indirect Nikolsky sign), where applying vertical pressure on a bulla causes it to spread to adjacent unaffected areas.^[32]^

Our review also revealed multiple erosive and ulcerative lesions as the primary manifestations, with the buccal mucosa, labial mucosa, tongue, and palate being the most commonly affected sites.^[6,14,20,22,23,27,28]^

Patients with JPV frequently report excessive salivation, bloody saliva, foul odor, and difficulties with speaking, chewing, and swallowing.^[11,33]^ In our review, Hasan et al documented a case of juvenile pemphigus vulgaris in a 16-year-old female patient who exhibited profuse salivation, difficulty in swallowing, and a positive Nikolsky sign.^[6]^

Gingival lesions are rare at onset and may present as isolated blisters or erosions, primarily affecting the free gingiva, making them challenging to identify as bullous lesions. However, advanced cases typically exhibit severe desquamative or erosive gingivitis.^[34]^ In our review, gingival hyperplasia was observed in a 17-year-old female patient.^[17]^

PV should be considered in cases of isolated oral erosions to prevent life-threatening complications. As oral lesions are often the 1st or only sign, dentists play a key role in early diagnosis and management. Delayed diagnosis is common in such cases, highlighting the importance of prompt recognition.^[31,35]^

JPV has a higher prevalence of genital and ocular lesions than the adult variant.^[29]^ JPV features unpredictable clinical progression, yet it is typically regarded as more favorable than that in adults.^[6,7,15]^

Clinical presentation, histopathological evaluation, and demonstration of tissue-bound and/or circulating autoantibodies form the diagnostic cornerstones for PV.^[36,37]^ The diagnostic tests for PV are identical for both adult and pediatric patients, as they display comparable clinical, histological, and IF characteristics.^[22]^

In our review, the diagnosis was confirmed using HP, IF, and ELISA in 23 patients,^[6,16–18,21,24–26]^ HP and IF in 8 patients,^[12,14,23]^ and a combination of TS and HP in 2 patients.^[19]^ Additionally, the diagnosis was based solely on HP in 2 patients,^[27,28]^ IF in 1 patient,^[20]^ and TS in 1 patient.^[22]^

The goal of PV treatment is to achieve and maintain remission, marked by the cessation of new blister formation, healing of existing erosions, and successful tapering to a maintenance dose. The primary challenge thereafter is preventing long-term relapse while minimizing adverse effects associated with prolonged CS and immunosuppressive therapy.^[38]^

Different associations have established many guidelines and consensus statements related to the management of pemphigus. The treatment approach for PV differs across these guidelines, highlighting the disease’s complexity and the diverse treatment options available.^[39]^

The Association of the Scientific Medical Societies in Germany recommends sCS as the 1st-line treatment for moderate to severe pemphigus, in combination with immunosuppressants from the outset. The initial dosage of sCS typically ranges from 1.0 to 1.5 mg/kg/d. Pulsed intravenous CSs are also considered a viable alternative 1st-line therapy for moderate to severe cases.^[40]^

CS dose may be increased to a maximum of 2 mg/kg/d in recalcitrant cases. For patients receiving only sCS, adjuvant medications should be added, while those already on immunosuppressive therapy should be switched to an alternative adjuvant drug.^[40]^ The guidelines recommend increasing the CS dose and evaluating the need for additional cycles of RTX in cases of relapse.^[39]^

The Brazilian Society of Dermatology recognizes sCS as the 1st-line treatment for all severity levels, with an initial dosage of 0.5 to 1.0 mg/kg/d. While recommending the use of immunosuppressants alongside CS, the society emphasizes CS therapy as the key component of initial treatment.^[41]^

The British Association of Dermatologists advocates sCS as the primary treatment, starting with a dose of 0.5 to 1.0 mg/kg/d, alongside immunosuppressants from the outset of therapy.^[40]^ The CS dose may be increased up to 2 mg/kg/d if the lesions do not resolve within 1–2 weeks. CS therapy is continued until 80% of lesions have healed and no new lesions develop for at least 2 weeks, followed by a gradual dose tapering to minimize side effects. Adjuvant immunosuppressants like Aza and MMF improve treatment efficacy and reduce CS dependence, while RTX shows higher complete remission rates than CS alone.^[42]^

The European Academy of Dermatology and Venereology guidelines recommend RTX with two 1 g infusions given 2 weeks apart, combined with sCS (prednisone at 1 mg/kg/d), followed by a gradual taper over approximately 6 months. RTX can be used alongside topical CS when sCS are contraindicated. If RTX is not suitable, oral prednisone and other immunosuppressive agents may be used as alternatives.^[38]^

The French Society of Dermatology advocates topical CS as the primary treatment for localized or mild PV, whereas sCS (initial dose of 1.0 to 1.5 mg/kg/d) are recommended for more widespread cases.^[43]^ The guidelines highlight the cautious use of CS therapy to minimize adverse effects, recommending a gradual tapering strategy for long-term management. RTX may be considered for patients who are CS-resistant or prone to frequent relapses, offering a targeted treatment approach.^[39]^

The Dermatology Branch of China International Exchange and Promotion Association for Medical and Healthcare recommends sCS (initial dose of 1.0 mg/kg/d) as the primary treatment and advocates early use of immunosuppressants such as Aza, MMF, or Cyp to reduce CS dosage and long-term risks. RTX is advised for patients who do not respond sufficiently to CS alone or who experience frequent relapses.^[44]^

The combination of sCS (prednisone/prednisolone; 1.0–1.5 mg/kg/d) and potentially CS-sparing immunosuppressive drugs, mostly Aza and MMF, was regarded as standard 1st-line therapy by most clinicians.^[38]^ Generally, mild pemphigus treatment involves lower steroid doses compared to moderate/severe disease treatment, which involves higher steroid doses with the addition of steroid-sparing adjuvant agents.^[3]^

There are no Food and Drug Administration-approved treatment strategies for JPV because of the insufficient number of controlled trials in this population. Currently, no therapeutic guidelines have been established for this patient population.^[6,7,15]^ CSs are the mainstay of treatment for JVP. Various factors, such as age, weight, disease severity, and potential adverse drug effects, should be considered when determining the dose for JPV patients.^[6]^ Most practitioners consider the use of sCS in conjunction with CS-sparing immunosuppressive agents to be the cornerstone of treatment.^[38]^

The treatment protocols differed among the reviewed studies. The most frequently employed 1st-line strategy was CS combined with immunosuppressive agents (Dap, Aza, Cyp, and MMF). However, in 3 studies, CS alone was used to treat JPV.^[6,22,23]^

Prolonged steroid treatment may result in a range of local adverse effects (including mucosal atrophy, and oral candidiasis) and systemic complications (such as adrenocortical suppression, hypertension, hyperglycemia, psychosomatic ailments, and osteoporosis). Additionally, many patients were unresponsive to CSs and CS-sparing therapies, thus paving the way for the use of biologics such as RTX.^[45]^

In our review, RTX was used in several studies for cases refractory to CS, Aza, MMF, methylprednisolone (MP), and IV IG.^[12,14,16,18–21,24–28]^

In a study by Vinay K et al,^[18]^ RTX was used as a 2nd-line therapy to treat 5 patients with JVP. Two patients received RTX as they were refractory to Aza and CS, while 2 others with severe disease and unresponsive to Aza, CS, DP, and MMF, were also treated with RTX. One patient with severe disease, refractory to CS therapy and who developed iatrogenic Cushing’s syndrome was also treated with RTX.

In a study by Renert-Yuval et al,^[14]^ RTX was used as a 2nd-line therapy in 3 JVP patients who had previously received sCS for at least 1 year.

In a study by Bilgic-Temel et al,^[16]^ RTX was administered as a 2nd-line therapy to treat 3 JVP patients due to either treatment failure or severe adverse effects associated with MP, Aza, Dap, MMF, and IV IG.

RTX was employed as a 2nd-line therapy to treat 2 JVP patients in a case series by Buch et al.^[19]^ RTX was used due to either new lesion development despite therapy or severe adverse effects associated with Aza and dexamethasone.

In a retrospective study by Salman et al,^[12]^ complete remission was achieved following RTX infusions in 2 JVP patients. RTX was administered due to either treatment failure or severe adverse effects associated with MP, Aza, Dap, and IV IG.

In a study by Gupta et al,^[21]^ RTX was administered to 2 JVP patients who did not respond to CS, Aza, Cyp, or other treatment options.

RTX was also employed in many JVP case reports and case series, either with failed treatment with CS and other steroid-sparing agents or due to the adverse effects of multiple therapies.^[20,24–28]^

As shown in studies,^[12,14,18,19,21,24–27]^ RTX resulted in long-term remission without relapse. Haberland et al.^[17]^ performed surgical excision of hypertrophied gingival tissue in a 17-year-old patient.

Our review suggests that RTX can be considered as a 1st-line treatment for JPV patients. Notably, the long-term use of RTX in the pediatric population has demonstrated an overall acceptable safety profile, with no increase in adverse events, even with multiple treatment courses or higher cumulative doses.

The response to treatment varies significantly among patients, with frequent clinical relapses. Therefore, close clinical monitoring and regular assessment of anti-DSG1 and anti-DSG3 antibody levels are crucial, as their levels correlate with disease activity and help predict relapses.^[46]^

As ELISA serves as a reliable qualitative and quantitative method for detecting serological autoantibody titers, the correlation between anti-DSG titers and disease activity as well as severity has been extensively investigated.^[47]^

Studies suggest that a decline in autoantibody titers is associated with clinical remission, whereas persistently high or rising titers may indicate an increased risk of relapse.^[46]^ Higher anti-Dsg1 antibody levels are associated with increased severity of cutaneous lesions, while elevated anti-Dsg3 antibody levels correlate with the severity of mucosal involvement.^[48]^

The review is primarily based on case reports and studies with small sample sizes, resulting in limited robust evidence and making generalizations challenging due to variations in clinical presentations and treatment responses among patients. The absence of controlled clinical trials specifically designed for pediatric patients with PV further restricts the ability to draw definitive conclusions regarding treatment efficacy. Consequently, well-established treatment guidelines for this demographic are lacking. Additionally, inconsistencies in case definitions, diagnostic criteria, and treatment approaches across the included studies introduce variability, complicating direct comparisons and potentially obscuring key trends in JPV management.

These limitations emphasize the need for larger, controlled studies focused on juvenile populations to establish standardized treatment protocols and enhance understanding of JPV outcomes.

## 5. Conclusion

JPV is a rare autoimmune blistering dermatosis that presents considerable diagnostic and therapeutic challenges. Managing JPV is further complicated by the potential adverse effects of prolonged immunosuppressive therapy. The current treatment mainly consists of sCS and immunosuppressive agents, while emerging biologic therapies, such as RTX, show promise. Nevertheless, our understanding of JPV remains limited, relying largely on case reports and data extrapolated from adult studies. Thus, there is an urgent need for further research to develop effective treatment protocols and improve the long-term outcomes in the pediatric population.

## Author contributions

**Conceptualization:** Shyamkumar Sriram, Shamimul Hasan.

**Methodology:** Shyamkumar Sriram, Mambakkam Jayakanth, Tanveer Alam, Shazina Saeed, Shamimul Hasan.

**Validation:** Shyamkumar Sriram, Mambakkam Jayakanth, Tanveer Alam, Shazina Saeed, Shamimul Hasan.

**Writing – original draft:** Shamimul Hasan.

**Writing – review & editing:** Mambakkam Jayakanth, Tanveer Alam, Shamimul Hasan.
